# An identity threat perspective on why and when employee voice brings abusive supervision

**DOI:** 10.3389/fpsyg.2023.1133480

**Published:** 2023-06-07

**Authors:** Lei Wu, Anna Long, Chenbang Hu, Yunpeng Xu

**Affiliations:** ^1^Human Resource Management, School of Business, Shaoxing University, Shaoxing, China; ^2^Business Administration, School of Business, Jishou University, Jishou, China; ^3^Tourism Management, School of Business Administration, Zhongnan University of Economics and Law, Wuhan, China

**Keywords:** abusive supervision, employee voice, identity threat, supervisor traditionality, organizational management

## Abstract

**Purpose:**

Drawing from identity threat theory, this study aims to understand how and when employee voice can lead to abusive supervision. It proposes and examines a theoretical model in which employee voice is linked to abusive supervision through the mediating effect of leader identity threat.

**Methods:**

We conducted a field study by collecting data from 93 supervisors and 533 subordinates in China at two different points in time. A structural equation model and Mplus software were used to examine the direct relationship between employee voice and abusive supervision, as well as the mediating effect of leader identity threat and the moderating effect of supervisor traditionality.

**Results:**

Our results showed that employee voice was positively related to leader identity threat and had an indirect effect on abusive supervision via leader identity threat. In addition, we found that supervisor traditionality moderated the relationship between employee voice and leader identity threat. Subordinates’ voice increased perceptions of leader identity threat among supervisors with high traditionality, whereas supervisors with low traditionality did not make this association. Finally, the indirect effect of employee voice on abusive supervision via leader identity threat was moderated by supervisor traditionality.

**Discussion:**

First, this study broadens our understanding of the antecedents of abusive supervision by proposing that employee voice may induce abusive supervision. Second, it develops an identity threat perspective to explain why employee voice is positively related to abusive supervision. Finally, it enriches the research on implicit leadership theories by proposing that supervisors’ cultural values can also influence supervisors’ sense-making of subordinates’ behaviors.

## 1. Introduction

In organizational management, the role of the leader is considered important because leaders influence employees’ behavior and the work environment (Islam et al., 2018). The positive effects of leadership on organizations and employees have been widely discussed. In fact, researchers have found that the negative effects of leadership on the organization are often greater than the positive effects because those in leadership positions sometimes have the capacity and motivation to be destructive (Tierney and Tepper, 2007; Islam et al., 2021). Abusive supervision refers to “subordinates’ perceptions of the extent to which supervisors engage in the sustained display of hostile verbal and nonverbal behaviors, excluding physical contact (Islam et al., 2021).” It is an important antecedent to many negative consequences of leadership (Tepper, 2000), and can cause many deleterious outcomes to employees and organizations (Harakan et al., 2020), such as high job stress, high turnover intention, low self-efficacy, low performance, and knowledge hiding (for recent reviews, see Tepper et al., 2017; Fischer et al., 2021). As abusive supervision is a widespread problem in the workplace, scholars have long been concerned about its harmful consequences (Hoobler and Hu, 2013; Wheeler et al., 2013). Zhang and Liao (2015) integrated the research on the consequences and moderators of abusive supervision to construct a framework for a comprehensive and systematic understanding of the consequences of abusive supervision. However, although the literature has explored the negative consequences of abusive supervision in depth, this knowledge does not mitigate its negative effects. In response, Tepper (2007) suggested that one way to help us reliably prevent abusive supervision is to explore why and when leaders abuse their subordinates, and called for a shift from outcome studies of abusive supervision to research on antecedents.

Review of antecedents leading to the formation of abusive supervision. From the leadership perspective, research has found that supervisors with high levels of Machiavellianism (De Hoogh et al., 2021), narcissism (Waldman et al., 2018), creative mindset (Qin et al., 2020), or irritation (Pundt and Schwarzbeck, 2018) are more likely to abuse their subordinates. In addition, abusive supervision by supervisors’ leaders (Liu et al., 2012; Mawritz et al., 2012), work–family conflict (Courtright et al., 2016), perceived workplace competitiveness (Ng et al., 2021), and high performance work systems will also increase abusive supervision (Xi et al., 2021). For the subordinate-related factors, subordinates’ personality traits and their dissimilarity to supervisors’ personality traits may influence their supervisors’ abusive behavior (Tepper et al., 2011; Henle and Gross, 2014; Wang et al., 2015). Recently, organizational scholars have focused on subordinates’ performance and behaviors, finding that subordinates’ poor performance (Wang et al., 2015; Liang et al., 2016), interpersonal deviance (Eissa et al., 2020), and organizational deviance can all trigger leaders’ abusive behaviors (Liang et al., 2016). Employee behavior is one of the main reasons behind leaders’ abusive supervision (Zhang and Liao, 2015). However, researchers have largely looked at the antecedents that lead to abusive supervision from the perspective of employees’ negative behaviors, while ignoring the effects of employees’ positive behaviors on abusive supervision. This oversight hinders our full understanding of the antecedents of abusive supervision from the perspective of subordinate-related behaviors.

Employee voice is defined as employee’s expression of constructive opinions, concerns, or ideas about work-related issues, which is a positive behavior (Tangirala and Ramanujam, 2008). In the current organizational environment, which emphasizes flexible innovation and continuous improvement, more and more organizations are involving employees in workplace decision-making, and soliciting feedback (Budd et al., 2010). Employee voice can diagnose workplace problems and difficulties for the organization and provide sensible suggestions for improvement, helping the organization improve learning ability, enhance organizational activity, increase operational efficiency, and build core competitiveness. When suggestions are adopted, the job satisfaction (Nawakitphaitoon and Zhang, 2020), wellbeing (Avey et al., 2012), work creativity (Dedahanov et al., 2016), and work engagement of employees also increase (Ge, 2020). Even so, it remains uncertain whether employee voice leads to abusive supervision. Research has not yet fully answered this question. However, clarifying how and when supervisors respond negatively to employee voice can guide employees when they want to speak up. This is conducive to encouraging employee voice and promoting the development of the organization.

Drawing from the identity threat theory to explore the mechanisms between employee voice and abusive supervision, and utilize implicit leadership theory to examine the boundary conditions of them. Identity threat theory holds that status maintains a leader’s perception of self-worth. When status is challenged, the leader may perceive his or her identity to be under threat. To counter that perceived threat, leaders often adopt strategies designed to reshape and reinforce their identity (Cui et al., 2019). We propose that employee voice may threaten a supervisor’s identity as a leader by challenging their authority and competence, thus evoking supervisory abuse. At the same time, implicit leadership theory notes that leaders with different traits pay different amounts of attention to the value of status. A leader’s perception of any threat to their identity will be influenced by leadership traits, particularly in organizational contexts where leaders have a traditional view of hierarchy. Supervisors with high traditionality, who endorse the traditional hierarchical role relationships prescribed by Confucian social ethics (Farh et al., 2007), will perceive high levels of threat to their identity as a leader when their subordinates speak up.

**Figure 1:**
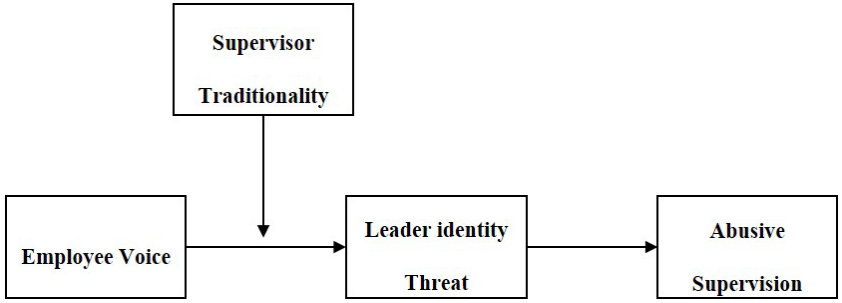
The theoretical model.

In summary, this article proposes a moderated mediation model (Figure 1) in which employee voice promotes abusive supervision *via* leader identity threat and argues that this indirect effect is contingent on supervisor traditionality. It contributes to the literature on abusive supervision in four ways. First, we examine a proactive behavior (i.e., employee voice) as an important antecedent of abusive supervision. The research has largely focused on abusive supervision triggered by employees’ negative behaviors, while our study proposes that positive behaviors may also induce abusive supervision. Second, we provide an identity threat perspective to explain why employee voice is positively related to abusive supervision. Although identity threat has been considered as an important mechanism linking antecedents with abusive supervision (Tepper et al., 2017), leader identity threat has not been examined empirically. Our study provides direct support for this mechanism. Third, our study enriches the research on implicit leadership theories by proposing that supervisors’ cultural values can influence the way in which supervisors make sense of subordinates’ behaviors (Epitropaki and Martin, 2004; Lord et al., 2020). Finally, the moderating role of supervisors’ traditionality in the relationship between employee voice and leader identity threat provides some insights into the employee voice literature. Research has found that supervisors may see employee voice as personally threatening (Burris, 2012), while our study proposes that supervisors’ threat perceptions will be lower when they have lower traditionality.

## 2. Theoretical development and hypotheses

### 2.1. Identity threat theory and leader identity threat

Identity threat theory consists of two main stages: threat generation and response (Petriglieri, 2011). The theory suggests that individuals have multiple identities in society and maintain a sense of self-worth through these identities (Reed, 2004). Usually, identity is a stable state in a given context and provides a sense of meaning (Erikson, 1968). When individuals perceive that something is hindering the expression of their identity (Elsbach, 2003), they are prone to self-doubt and, to a certain extent, perceive their identity as being threatened (Cui et al., 2019). When an identity threat arises, they will adopt coping methods to alleviate the unease and to repair the value and sense of belonging in that social identity, and reshape the integrity of the values shaping their identity.

In the workplace, because they occupy a formal role as leader, supervisors incorporate the leadership role into their self-definition, a step known as leader identity (Lord and Hall, 2005; DeRue and Ashford, 2010; Kwok et al., 2018). Although the role is granted by the institutional setting, supervisors’ leader identity is malleable and varies across different interactions with their subordinates (DeRue and Ashford, 2010). At work, supervisors can maintain, strengthen, and repair their leader identity *via* their identity-claiming (the actions that supervisors take to assert their identity as a leader) and subordinates’ identity-granting (the actions that subordinates take to bestow leader identity on the supervisor). When identity-claiming or identity-granting is hindered, supervisors may perceive their leader identity to be under threat, and perceptions of the value and enactment of leader identity may potentially be harmed (Petriglieri, 2011). Therefore, the threat to supervisors’ leadership identity can be activated by their perception of the difference between an ideal leadership identity and the leadership identity actually granted by employees (Higgins, 1987). For example, supervisors may perceive a leader identity threat when subordinates challenge their authority (Tedeschi and Felson, 1994; Tepper et al., 2017; Güntner et al., 2021). In the next section, we elaborate in detail on how and when employee voice, a challenging behavior, is positively related to leader identity threat.

### 2.2. Employee voice and leader identity threat

Employee voice is a challenging behavior meant to benefit the group or organization. Research has found that employee voice can lead to many positive outcomes, such as higher status quo-challenging performance evaluation of voicers (Grant, 2013), more team learning (Edmondson, 2003), and better group performance (Li et al., 2017). However, as employee voice reflects employees’ dissatisfaction with the status quo and their intention to challenge it, supervisors may feel leader identity threat for at least two reasons. First, for leaders, identifying the problems of their team is a core job for a leader. When employees speak up about problems in the group, it implies that the supervisor is ignorant of something important about their job. Preventing problems and enhancing the team’s performance are critical components of a leader’s role identity, so any problems with the team threaten a supervisor’s fitness to occupy a leadership role. Second, leader identity is socially constructed in the process of interaction between supervisors and their subordinates (DeRue and Ashford, 2010). Employee voice challenges supervisors’ authority over their team, and can threaten supervisors’ leader identity through the experience of a loss of power and agency. Research has also found that employee voice may increase supervisors’ perceptions of threat (Burris, 2012). Thus, we propose the following hypothesis:

*Hypothesis 1*: Employee voice is positively related to leader identity threat.

Implicit leadership theory suggests that leaders have different traits that may influence their cognition and behavior (Shondrick et al., 2010). In organizational situations, subordinates’ perception of compliance with authority is viewed as a traditional value by leaders, which can influence their perceptions and understanding of subordinates’ behavior (Offermann et al., 1994; Epitropaki and Martin, 2004; Lord et al., 2020). Related research suggests that supervisors with high traditionality, they value the extent to which supervisors and their subordinates fulfill responsibilities which defined by prescribed social roles (Farh et al., 1997). More importantly, these supervisors emphasize the hierarchical differences between themselves and their subordinates, and hence “expect followers to be told what to do, and avoid interacting with subordinates in an open manner (Wang and Kim, 2013).” When subordinates propose ideas to supervisors with high traditionality, these supervisors may feel that they have failed to claim their leader role, which, in turn, threatens their supervisory status. In contrast, supervisors with low traditionality do not consider subordinates’ conformity to be a successful enactment of their leader identity. Thus, we propose the following hypothesis:

*Hypothesis 2*: Supervisor traditionality moderates the relationship between employee voice and leader identity threat, such that this relationship is stronger when supervisor traditionality is high than when it is low.

### 2.3. Leader identity threat and abusive supervision

The second stage of identity threat theory is responding to the threat (Petriglieri, 2011). When supervisors feel that their leadership is threatened, they will adopt strategies to eliminate the threat and maintain leadership status, such as abuse of subordinates. Supervisors often act aggressively toward subordinates in an effort to confirm their authority and influence (Tedeschi and Felson, 1994). On the one hand, by abusing subordinates, supervisors can force subordinates to obey their orders, alleviating the threat to leader identity caused by subordinates’ nonconformity. On the other hand, supervisors can also maintain and reinforce their authority by abusing the subordinates who are challenging their image of hierarchical structure (Tedeschi and Felson, 1994). Research has found that supervisors are more likely to abuse subordinates who engage in organizational deviance (Lian et al., 2014) and supervisor-directed avoidance (Simon et al., 2015). In addition, Khan et al. (2018) found that supervisors with high social dominance orientation increasingly abused high-performing subordinates as their perceptions of a threat to hierarchy increased. This suggests that leader identity threat caused by employee voice is positively related to abusive supervision, leading to our third hypothesis:

*Hypothesis 3*: Leader identity threat is positively related to abusive supervision.

As mentioned above, employee voice may be perceived as a behavior that challenges the leadership role, thus causing leadership identity threat. Leaders who experience a sense of threat are likely to practice abusive supervision, so we further propose the following hypothesis:

*Hypothesis 4*: Employee voice is positively related to abusive supervision via leader identity threat.

Combining *Hypothesis 2 and 4*, we also propose a moderated mediation hypothesis:

*Hypothesis 5*: Supervisor traditionality moderates the indirect relationship between employee voice and abusive supervision via leader identity threat, such that this indirect relationship is stronger when supervisor traditionality is high than when it is low.

## 3. Method

### 3.1. Procedure and sample

We collected Stata using questionnaires in a large company in China with more than 4,000 employees. The first author contacted the company’s executives to explain the purpose of our research and received permission to distribute questionnaires in the company. We selected 100 teams with more than three employees to participate in our study. To reduce the possibility of common method bias, there were two data collection phases. At Time 1, we asked supervisors to rate their traditionality and demographic information. We also asked subordinates to rate their voice and demographic information. After participants filled out the questionnaires, we gave each participant an envelope (each bearing a unique code [e.g., ZJ00101] shared by participants in the same team [e.g., ZJ00101, ZJ00102, ZJ00103]) and asked them to put the questionnaire into the envelope and seal it. We then collected the first envelopes, and simultaneously gave each participant a new sealed envelope with the same code as the previous envelope. They were told that they were not allowed to open these envelopes and that we would have another meeting with them the following month. To participate in the following month’s meeting, they were told that they should bring the envelopes with them. One month later (Time 2), we gathered these supervisors and subordinates in a large conference room and asked them to open their envelopes and fill out the questionnaires. This time, supervisors were asked to rate their leader identity threat, and subordinates were asked to rate their supervisors’ abusive supervision.

At Time 1, 100 supervisors and 653 subordinates filled in the questionnaires. At Time 2, there were 93 supervisors (response rate = 93.0%), and 533 subordinates who filled in the questionnaires (response rate = 81.6%). Among the 93 supervisors, 80.0% were male, the mean age was 38.2 years (SD = 8.18 years), the mean organizational tenure was 12.0 years (SD = 9.58 years), and 51.8% had a Bachelor’s degree. Among the 533 subordinates, 71.7% were male, the mean age was 34.9 years (SD = 8.67 years), the mean organizational tenure was 9.1 years (SD = 8.86 years), and 43.9% had a Bachelor’s degree.

### 3.2. Measures

All measures used 7-point scales (1 = strongly disagree to 7 = strongly agree) and were translated to Chinese following the method recommended by Brislin (1986).

*Employee voice*: We measured employee voice (α = 0.89) using the 6-item scale developed by Van Dyne and LePine (1998). Employees were asked to report on their agreement with statements about team members’ behaviors. A sample item is “Group members are kept well informed about issues where they opinion might be useful to this work group.”

*Traditionality*: We measured leaders’ traditionality (α = 0.85) using the 5-item scale developed by Farh et al. (1997). Leaders were asked to report their agreement with statements on their values such as “The chief government official is like the head of a household, the citizen should obey his decisions on all state matters.”

*Leader identity threat*: We measured leader identity threat (α = 0.93) using a 4-item scale reported by Greenbaum et al. (2021), which was originally developed by Aquino and Douglas (2003). Leaders were asked to report their agreement with statements on their perceptions such as “My role as a leader was looked at in a negative way.”

*Abusive supervision*: We measured abusive supervision (α = 0.97) using the 5-item scale developed by Mitchell and Ambrose (2007). Subordinates were asked to report their agreement with statements about their supervisors’ behaviors. A sample item is “My supervisor tells me my thoughts or feelings are stupid.”

*Control variables*: To enhance the robustness of our results, we also reran our model while controlling for leaders’ age, gender, organizational tenure, and the mean of team members’ age and organizational tenure. The results did not change the conclusions drawn from the model’s results without the controls. For parsimony, we report the model’s results without the control variables.

## 4. Results

We adopted structural equation modeling for our analysis, a technique that is suitable for analyzing causal relationships between variables according to a specific working model (Bielby and Hauser, 1977). For the data analysis, we adopted Mplus 8.0, a powerful data processing software package that has been widely used in empirical research (Deng et al., 2022). The data analysis was divided into two stages (Islam et al., 2022a,b). In the first stage, we used confirmatory factor analyses to assess the measurement model and, in the second stage, when the model had good discriminative validity (Duan et al., 2020), we tested our hypotheses.

### 4.1. Confirmatory factor analyses

Before testing the hypotheses, we ran a series of multilevel confirmatory factor analyses using Mplus 8.0 to examine the distinctiveness of our main variables. The model fitness indices were Chi-square/degrees of freedom (*χ*^2^/*df* ≤ 3.0), root mean square error of approximation (RMSEA ≤ 0.08), comparative fit index (CFI ≥ 0.90), and Tucker–Lewis Index (TLI ≥ 0.90) (Marsh et al., 1988; Hevey et al., 2010). The results showed that the four-factor model was a good fit (*χ*^2^/*df* = 2.36, RMSEA = 0.05, CFI = 0.94, TLI = 0.93) and was better than two three-factor models: the model combining leader identity threat with traditionality (*χ*^2^/*df* = 3.43, RMSEA = 0.07, CFI = 0.90, TLI = 0.88) and the model combining employee voice with abusive supervision (*χ*^2^/*df* = 9.37, RMSEA = 0.13, CFI = 0.65, TLI = 0.59). These results indicated a good discriminatory validity among the variables of our model.

### 4.2. Data aggregations

As our theory applies to the level of the team level, we aggregated employee voice and abusive supervision at the team level. To examine the appropriateness of these aggregations, we calculated the intra-class correlation coefficients (i.e., ICC[1] and ICC[2]) and within-group inter-rater reliabilities (i.e., Rwg). The results showed that our aggregations were appropriate: Employee voice, ICC[1] = 0.20, ICC[2] = 0.58, Rwg[mean] = 0.94, Rwg[median] = 0.96; abusive supervision, ICC[1] = 0.26, ICC[2] = 0.66, Rwg[mean] = 0.93, Rwg[median] = 0.96.

### 4.3. Hypothesis tests

Table 1 presents the descriptive statistics and correlations among our main variables. Hypothesis 1 proposed that employee voice is positively related to leader identity threat. To test this hypothesis, we ran a mediation model (Model 1) that controlled for the direct effect of supervisor traditionality. As is shown in Table 2 (Model 1), we found that employee voice was positively related to leader identity threat (*B* = 0.42, *p* < 0.001). Hence, Hypothesis 1 was supported.

**Table 1:** Descriptive statistics and correlations among main variables.

Variables	M	SD	1	2	3	4
**Subordinate level**						
1. Employee voice	5.78	0.88	—			
2. Abusive supervision	3.86	1.03	0.24**	—		
**Supervisor level**						
3. Leader identity threat	4.15	0.61	0.35**	0.43**	—	
4. Supervisor traditionality	3.20	0.81	0.003	0.07	0.01	—

*n* = 93 supervisors, *N* = 533 employees. The aggregations of subordinate-level variables’ scores were used to calculate their correlations with supervisor-level variables.

** *p* < 0.01.

**Table 2:** Results of analyses.

Variables	Model 1		Model 2	
	1	2	1	2
	B (SE)	B (SE)	B (SE)	B (SE)
Employee voice	0.42^**^ (0.12)	0.12 (0.15)	0.31^*^ (0.12)	0.14 (0.16)
Leader identity threat		0.60^***^ (0.14)		0.63^***^ (0.14)
Supervisor traditionality	0.01 (0.08)	0.04 (0.09)	−0.13 (0.08)	0.08 (0.10)
Employee voice*supervisor traditionality			0.56^**^ (0.17)	−0.15 (0.21)

*n* = 93 supervisors, 1 = Leader identity threat, 2 = Abusive supervision. ^*^p < 0.05, ^**^p < 0.01, ^***^p < 0.001.

**Figure 2:**
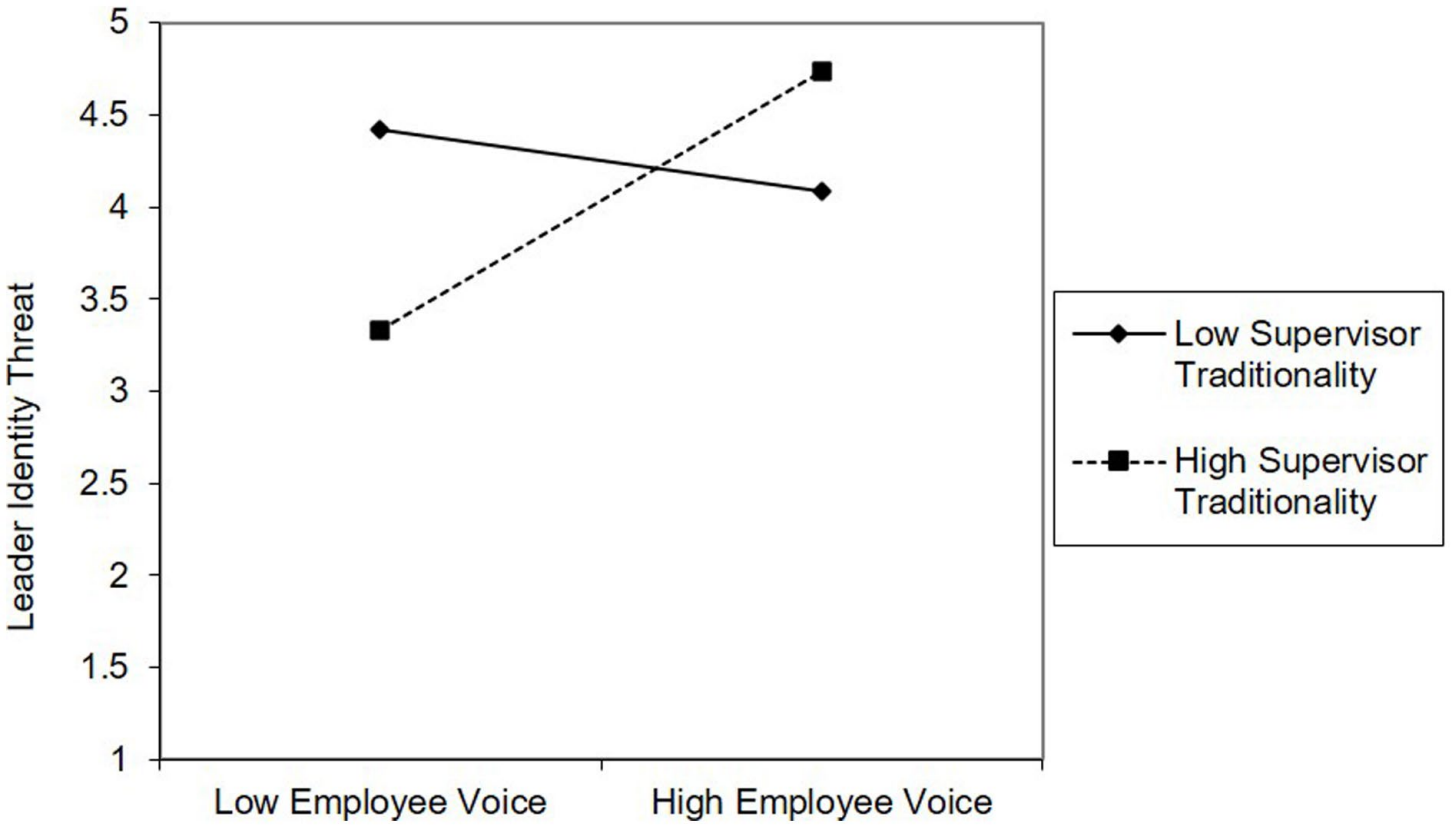
The moderating role of supervisor traditionally in the relationship between employee voice and leader identity threat.

Hypothesis 2 suggested that supervisor traditionality moderates the relationship between employee voice and leader identity threat, such that this relationship is stronger when supervisor traditionality is high rather than low. To test this hypothesis, we ran another moderated mediation model (Model 2) by adding the interaction term (employee voice*supervisor traditionality) to Model 1. As is shown in Table 2 (Model 2), we found that the interaction term is positively related to leader identity threat (*B* = 0.56, *p* < 0.01). Figure 2 presents this interaction: when supervisor traditionality was high, the relationship between employee voice and leader identity threat was significantly positive (*B* = 0.76, *p* < 0.001), whereas when supervisor traditionality was low, this relationship was not n-significant (*B* = −0.15, *p* > 0.47). These results supported *Hypothesis 2*.

Hypothesis 3 proposed that leader identity threat is positively related to abusive supervision. As is shown in Table 2 (Model 1), we found that leader identity threat is positively related to abusive supervision (*B* = 0.60, *p* < 0.001). Hence, *Hypothesis 3* was supported.

Hypothesis 4 proposed that employee voice is positively related to abusive supervision *via* leader identity threat. To test this indirect effect, we employed a bootstrapping method with 10,000 replications for Model 1. The results showed that the indirect effect of employee voice on abusive supervision *via* leader identity threat was 0.25, 95% CI = (0.004, 0.672). Thus, *Hypothesis 4* was supported.

*Hypothesis 5* proposed that supervisor traditionality moderates the indirect relationship between employee voice and abusive supervision *via* leader identity threat, such that this indirect relationship is stronger when supervisor traditionality is high than when it is low. To test this indirect effect, we again employed a bootstrapping method with 10,000 replications for Model 2. The results show that the indirect effect of employee voice on abusive supervision *via* leader identity threat was stronger (the difference is 0.57, 95% CI = [0.134, 1.502]) when supervisor traditionality was high (the indirect effect is 0.48, 95% CI = [0.101, 1.120]) than when supervisor traditionality was low (the indirect effect is −0.09, 95% CI = [−0.608, 0.145]). Thus, *Hypothesis 5* was supported.

## 5. Discussion

By collecting multi-source and multi-time data, this study examined the effects of employee voice on abusive supervision *via* leader identity threat, as well as the moderating role of supervisor traditionality. The results of our analyses showed that employee voice was positively related to leader identity threat, and this relationship was strengthened by supervisor traditionality. In particular, for supervisors with high traditionality, their subordinates’ voice increased the supervisors’ perceptions of leader identity threat, whereas for supervisors with low traditionality, their subordinates’ voice was not related to their perceptions of leader identity threat. In addition, we found that leader identity threat was positively related to abusive supervision, and employee voice had an indirect effect on abusive supervision *via* leader identity threat. Finally, this indirect effect was also strengthened by supervisor traditionality. For supervisors with high traditionality, this indirect effect was stronger than the indirect effect for supervisors with low traditionality.

### 5.1. Theoretical implications

This study contributes to the literature on abusive supervision in four ways. First, our study shows that employee voice has a positive relationship with abusive supervision *via* leader identity threat, which enriches our understanding of the antecedents of abusive supervision. The prior research has largely focused on the negative effects of employees’ negative behaviors (e.g., organizational deviance and interpersonal avoidance) on abusive supervision, while our study proposes that positive behavior can also induce abusive supervision. This is consistent with the findings of Khan et al. that high performance by subordinates may also bring about abusive supervision (Khan et al., 2018). These results give us a more comprehensive understanding of the causes of abusive supervision.

Second, our study theorized, and examined, an identity threat mechanism to explain why employee voice is positively related to abusive supervision. Although prior research has theorized about the relationship between subordinates’ performance/behaviors and abusive supervision from the perspective of an identity threat (for a review, see Tepper et al., 2017), very few studies have provided direct empirical evidence for this mechanism. Our field study, with its multi-time and multi-source field data, provides direct support for this mechanism.

Third, our study shows that supervisors’ traditionality serves as a critical boundary condition in the relationship between employee voice and leader identity threat, which broadens the research on implicit leadership theories. These theories propose that supervisors’ implicit beliefs about the characteristics of an ideal leader (e.g., sensitivity, intelligence, and dedication) play an important role in the process through which supervisors make sense of subordinates’ behaviors (Epitropaki and Martin, 2004; Lord et al., 2020). Our findings enrich these theories by bringing in supervisors’ traditionality, thus providing a cultural value that can also influence supervisors’ perceptions and understanding of subordinates’ proactive behaviors.

Finally, the moderating role of supervisors’ traditionality in the relationship between employee voice and leader identity threat also contributes to the voice literature by pointing that supervisors’ values play a critical role in determining the risks of employee voice. Prior research has found that supervisors see employee voice as personally threatening (Burris, 2012), while our study proposes that supervisors’ threat perceptions will be lower when they have lower traditionality.

### 5.2. Managerial implications

Our study has important managerial implications. First, from the supervisor’s perspective, managers should be aware that they may feel threatened when their subordinates engage in challenging behaviors even these behaviors are beneficial to the organization. Managers influenced by the sense of threat are prone to abusive supervision, which not only affects employees’ motivation and triggers deviant behavior but also inhibits the frequency and the willingness with which employees suggest ideas. This is not conducive to organizational stability and development. Given that employee voice is associated with many positive outcomes, companies can generally provide managers with training courses on the benefits of employee voice to help them handle the issue of threatened leader identity, increase the value placed on employee voice, and take the lead in creating a workplace climate where employee voice is valued. In addition, a monitoring mechanism can be established for regulating managerial behavior to reduce abusive supervision by managers as a result of employee voice.

Second, this study found that the more managers valued the traditional roles and relationships between superiors and subordinates, the more those managers would perceive employee voice as a threat, thus increasing the likelihood of abusive supervision. Therefore, companies cannot ignore the issue of managers’ traditionality. For managers with high traditionality, organizations need to strengthen targeted coaching and training so that those supervisors establish a correct understanding of the relationship between the leader and subordinate and change some of their implicit beliefs on employees’ proactive behaviors.

Third, from the subordinate’s perspective, our findings remind subordinates that their challenging ideas may bring about abusive supervision, especially when interacting with supervisors with higher traditionality. Employees need training on how communication between supervisors and subordinates works. For example, when subordinates are less expert (i.e., have low credibility), they should make their point in an indirect and respectful manner (Lam et al., 2019). However, when a supervisor has lower traditionality, the employee can be more direct and effective. More interactive activities between supervisors and subordinates can also be carried out to boost levels of trust, reducing the threat caused by employee voice.

### 5.3. Limitations and directions for future research

Despite the strengths of our study, several limitations remain that need to be noted for future research. First, in addition to employee voice, staff often engage in proactive behaviors (e.g., taking charge, job crafting, issue selling, etc.) at work (Parker and Collins, 2010). It remains uncertain whether these positive behaviors lead to abusive supervision. Future studies can examine the potential relationship, as well as the different effects of various proactive behaviors on abusive supervision compared with employee voice.

Second, this research only reveals the boundary role of supervisor traditionality between employee voice and leader identity threat. However, according to implicit leadership theory (Offermann et al., 1994), supervisors may hold different implicit beliefs on the characteristics of an ideal leader, such as the 59 implicit traits classified by Lord et al. (1984). These implicit beliefs can also serve as important theoretical boundary conditions for the relationship between employee voice and leader identity threat. Thus, future research can also theorize and examine the moderating role of different types of implicit leadership beliefs.

Finally, although the multi-time and multi-source design of our field study can reduce common method bias (Podsakoff et al., 2003), the data were obtained by cross-sectional and subjective reporting and cannot provide causal evidence for our hypotheses (Evans, 1985). Future studies can employ experiments or longitudinal designs to replicate our theoretical model.

## Data availability statement

The raw data supporting the conclusions of this article will be made available by the authors, without undue reservation.

## Ethics statement

The studies involving human participants were reviewed and approved by The Ethics Committee of Shaoxing University. The patients/participants provided their written informed consent to participate in this study.

## Author contributions

LW, AL, CH, and YX contributed to the conception and design of the study. LW organized the database and wrote the first draft of the manuscript. AL performed the statistical analysis. LW, AL, and CH wrote sections of the manuscript. All authors contributed to the article and approved the submitted version.

## Funding

This research was supported by Major projects of National Social Science Foundation of China Study and explained the spirit of the Fifth Plenary Session of the 19th CPC Central Committee (grant no. 21ZDA028)” Construction of ecological friendly water environment management system in new rural areas of socialist modernisation pilot area, the Education Scientific Research Program of Zhejiang Province (grant no. Y202044572), Shaoxing University Annual Key Scientific Research Project (grant no. 2019SK001) “Research on the Incentives, Influence Effects and Intervention Mechanisms of Overqualification.”

## Conflict of interest

The authors declare that the research was conducted in the absence of any commercial or financial relationships that could be construed as a potential conflict of interest.

## Publisher’s note

All claims expressed in this article are solely those of the authors and do not necessarily represent those of their affiliated organizations, or those of the publisher, the editors and the reviewers. Any product that may be evaluated in this article, or claim that may be made by its manufacturer, is not guaranteed or endorsed by the publisher.
